# Severe Hematuria and Prevesical Hematoma after Open Ureterocystoneostomy: Sequel of Undiagnosed Hemophilia

**DOI:** 10.21699/ajcr.v7i5.486

**Published:** 2016-11-01

**Authors:** Eiji Hisamatsu, Kaoru Yoshino

**Affiliations:** Department of Urology, Aichi Children's Health and Medical Center, Japan

**Dear Sir,**

Surgical interventions for vesicoureteral reflux (VUR) include open or laparoscopic ureterocystoneostomy (UCN) and endoscopic injection. Historically, open UCN has been the gold standard in surgical treatment of VUR with high success and low complication rates. [1] Although open UCN causes bleeding from the incised bladder wall or dissected trigone, the bleeding is usually not severe unless a bleeding disorder is present. We report a case of bilateral VUR that developed severe persistent hematuria and prevesical hematoma after open UCN.

A 7-year-old boy was referred for management of febrile urinary tract infection (UTI). He had a history of recurrent UTIs during infancy. Voiding cystourethrography showed bilateral grade 4 VUR. Dimercaptosuccinic acid (DMSA) renal scintigraphy revealed bilateral renal scarring. Subsequently, bilateral UCN by the Cohen technique was performed. The postoperative course was uneventful till postoperative day (POD) 5. He developed fever and severe gross hematuria after removal of the urethral catheter. Ultrasonography showed a hematoma in the prevesical space. Even after the urethral catheter was reinserted, severe hematuria persisted for 4 days and the prevesical hematoma further increased in size. We performed further coagulation testing because preoperative coagulation testing showed slightly prolonged activated partial thromboplastin time (APTT) of 45 seconds (normal range, 30–40 seconds). The factor VIII activity was 22% (normal range, 62–145%), while von Willebrand activity/antigen were normal. The result led to a diagnosis of mild hemophilia A. On further interview, his parents recalled that he had developed persistent bleeding after tooth extraction. Additionally, when his little brother (3 years old) underwent inguinal herniorrhaphy, preoperative coagulation testing also showed a slightly prolonged APTT but he did not develop any would bleeding or hematoma. The little brother was also diagnosed with mild hemophilia A afterward. Gross hematuria subsided spontaneously, and the urethral catheter was removed on POD 20. After removal of the urethral catheter, gross hematuria did not recur and the prevesical hematoma did not increase in size. He was discharged on POD 25. Ultrasonography performed at 3-month after discharge showed complete resolution of prevesical hematoma.

An abnormal APTT or history of abnormal bleeding may lead to preoperative diagnosis of congenital bleeding disorders. On the other hand, a normal APTT with no family or personal history cannot always exclude the potential of congenital bleeding disorders including hemophilia. According to Teruya’s report, an APTT was normal even when factor VIII activity was 30%. When APTT was prolonged to more than the normal upper limit, factor VIII activity was 13%. He concluded that a normal APTT did not guarantee adequate coagulation factor levels. [2] Hemophilia A is graded into varying levels of severity depending on factor VIII activity (severe, less than 1%; moderate, 1–5%; mild, 5–40%). The factor VIII activity of our patient was 22%. It led to the diagnosis of “mild” hemophilia A. Patients with mild hemophilia may not bleed excessively until they experience trauma or surgery. [3] A more meticulous inquiry regarding bleeding tendency¬ is suggested in case of even slight abnormality of clotting profile or history of prolonged bleeding to preempt postoperative bleeding complications.

## Footnotes

**Source of Support:** Nil

**Conflict of Interest:** None declared

